# Intra-coronary imaging and transcriptomics of calcified nodules before and after intensive lipid-lowering therapy: a YELLOW III substudy

**DOI:** 10.1093/ehjimp/qyag092

**Published:** 2026-06-19

**Authors:** Pruthvi C Revaiah, Yuliya Vengrenyuk, JiaJia Liu, Keisuke Yasumura, Vemuri Krishna Santosh, Monika Karki, Ishani Bansal, Amit Hooda, Joseph K Sweeny, Sahil Khera, Vishal Kapur, Prakash Krishnan, Pedro R Moreno, Roxana Mehran, Xiaobo Zhou, Jagat Narula, Samin K Sharma, Annapoorna S Kini

**Affiliations:** Division of Interventional Cardiology, Ichan School of Medicine at Mount Sinai and Fuster Heart Hospital at Mount Sinai, One Gustave L. Levy Place, Box 1030, New York, NY 10029, USA; Division of Interventional Cardiology, Ichan School of Medicine at Mount Sinai and Fuster Heart Hospital at Mount Sinai, One Gustave L. Levy Place, Box 1030, New York, NY 10029, USA; University of Texas Health Sciences Center, Houston, TX, USA; Division of Interventional Cardiology, Ichan School of Medicine at Mount Sinai and Fuster Heart Hospital at Mount Sinai, One Gustave L. Levy Place, Box 1030, New York, NY 10029, USA; Division of Interventional Cardiology, Ichan School of Medicine at Mount Sinai and Fuster Heart Hospital at Mount Sinai, One Gustave L. Levy Place, Box 1030, New York, NY 10029, USA; Division of Interventional Cardiology, Ichan School of Medicine at Mount Sinai and Fuster Heart Hospital at Mount Sinai, One Gustave L. Levy Place, Box 1030, New York, NY 10029, USA; Division of Interventional Cardiology, Ichan School of Medicine at Mount Sinai and Fuster Heart Hospital at Mount Sinai, One Gustave L. Levy Place, Box 1030, New York, NY 10029, USA; Division of Interventional Cardiology, Ichan School of Medicine at Mount Sinai and Fuster Heart Hospital at Mount Sinai, One Gustave L. Levy Place, Box 1030, New York, NY 10029, USA; Division of Interventional Cardiology, Ichan School of Medicine at Mount Sinai and Fuster Heart Hospital at Mount Sinai, One Gustave L. Levy Place, Box 1030, New York, NY 10029, USA; Division of Interventional Cardiology, Ichan School of Medicine at Mount Sinai and Fuster Heart Hospital at Mount Sinai, One Gustave L. Levy Place, Box 1030, New York, NY 10029, USA; Division of Interventional Cardiology, Ichan School of Medicine at Mount Sinai and Fuster Heart Hospital at Mount Sinai, One Gustave L. Levy Place, Box 1030, New York, NY 10029, USA; Division of Interventional Cardiology, Ichan School of Medicine at Mount Sinai and Fuster Heart Hospital at Mount Sinai, One Gustave L. Levy Place, Box 1030, New York, NY 10029, USA; Division of Interventional Cardiology, Ichan School of Medicine at Mount Sinai and Fuster Heart Hospital at Mount Sinai, One Gustave L. Levy Place, Box 1030, New York, NY 10029, USA; Division of Interventional Cardiology, Ichan School of Medicine at Mount Sinai and Fuster Heart Hospital at Mount Sinai, One Gustave L. Levy Place, Box 1030, New York, NY 10029, USA; University of Texas Health Sciences Center, Houston, TX, USA; University of Texas Health Sciences Center, Houston, TX, USA; Division of Interventional Cardiology, Ichan School of Medicine at Mount Sinai and Fuster Heart Hospital at Mount Sinai, One Gustave L. Levy Place, Box 1030, New York, NY 10029, USA; Division of Interventional Cardiology, Ichan School of Medicine at Mount Sinai and Fuster Heart Hospital at Mount Sinai, One Gustave L. Levy Place, Box 1030, New York, NY 10029, USA

**Keywords:** calcified nodule, optical coherence tomography (OCT), near infrared spectroscopy-intravascular ultrasound (NIRS–IVUS), fibrous cap thickness (FCT), lipid core burden index (LCBI), transcriptomics

## Abstract

**Aims:**

Calcified nodules (CNs) contribute to 3–5% of acute coronary syndromes (ACS). Identifying peri-calcific lipid-rich areas is challenging with conventional imaging but feasible using near-infrared spectroscopy (NIRS). This YELLOW III substudy investigated the impact of maximal lipid-lowering on CN morphology and lipid content over 26 weeks.

**Methods and results:**

Patients with stable CAD and lipid-rich, non-obstructive plaques (lipid arc >90°, FCT <120 µm on OCT) received Evolocumab (140 mg biweekly) plus maximally tolerated statins. Lesions were evaluated at baseline and 26 weeks using combined NIRS–IVUS and OCT. Exploratory endpoints were the maximum 4 mm lipid core burden index (LCBI) in segments containing CNs (maxLCBI_4mm-CN_) and morphological changes in CN. Linear mixed-effects models were used to evaluate temporal changes and group interactions. Peripheral blood mononuclear cell (PBMC) transcriptomics were analysed. Among 110 patients, 43 (39.1%) had CNs. A total of 73 paired CNs were analysed for morphology, of which 65 CNs were ≥4 mm. CNs with lipid signal (CN_LS_) demonstrated a significant reduction in maxLCBI_4mm-CN_ from 176.8 to 113.3 (*β* = −63.51 ± 17.63; *P* < 0.001), whereas no change was observed in dense CNs (*P* = 0.317). Morphological parameters remained largely unchanged in both groups. A modest increase in the surrounding calcium arc was observed in CN_LS_ (*P* = 0.018), while depth showed a differential change between groups (*p*_interaction_ = 0.012). Transcriptomic analysis demonstrated baseline enrichment of neutrophil degranulation pathways in CN patients, which diminished at follow-up.

**Conclusion:**

Intensive lipid-lowering significantly reduces peri-calcific lipid burden without measurable short-term changes in CN morphology. The clinical implications of LCBI reduction in CNs warrant further investigation.

**Summary:**

Calcified nodules (CNs) are high-risk coronary features responsible for a significant portion of acute coronary syndromes, yet their response to intensive medical therapy remains poorly understood. This YELLOW III substudy utilized multi-modality intravascular imaging (NIRS–IVUS and OCT) to investigate the impact of 26 weeks of Evolocumab and maximally tolerated statins on CN morphology. In 43 patients with identified CNs, intensive lipid-lowering therapy led to a significant reduction in the maxLCBI_4mmCN_. Morphological parameters of CNs remained largely unchanged except for a modest increase in the surrounding calcium arc. Simultaneously, PBMC transcriptomic analysis revealed a marked attenuation of the neutrophil degranulation pathway, which was highly enriched at baseline. While the therapy successfully reduced the systemic inflammatory profile, a transition towards coagulation-related pathways at follow-up suggests a shift in the residual risk profile. These findings provide a novel mechanistic basis for using PCSK9 inhibitors to stabilize calcium-related high-risk plaques. Collectively, the data demonstrate that aggressive lipid-lowering not only modifies plaque architecture but also shifts the systemic biological environment from an inflammatory to a more stable one.

## Graphical Abstract

**Graphical Abstract:**
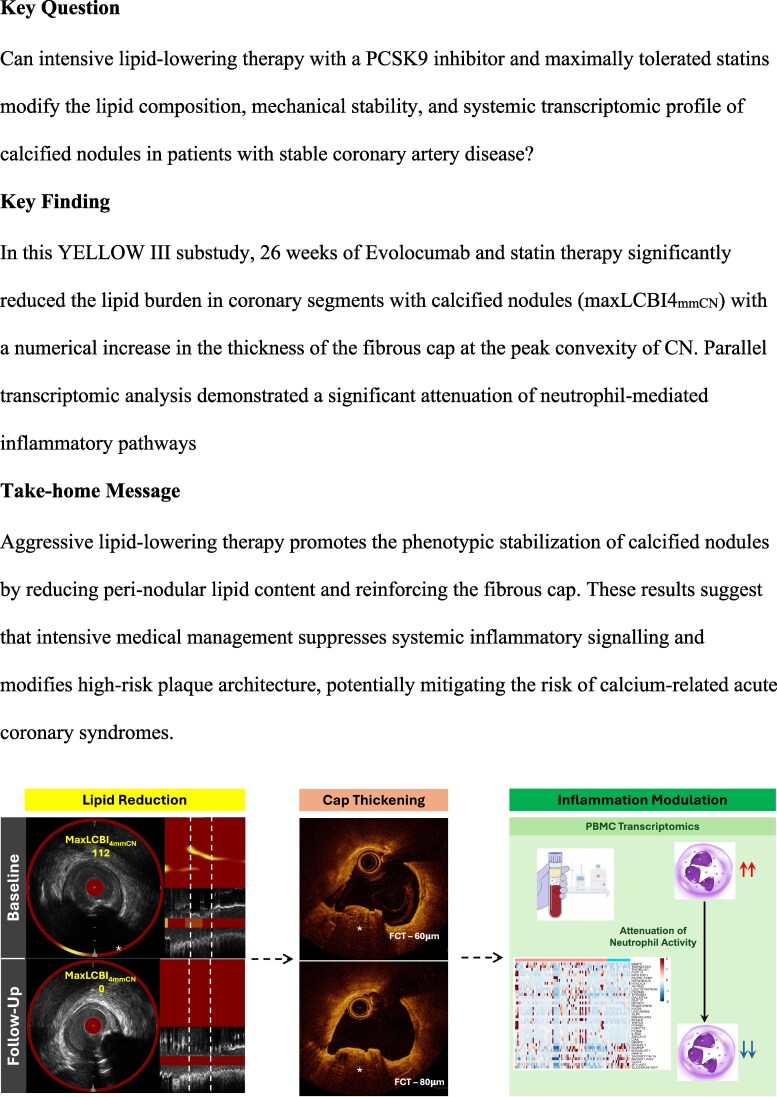
**Multimodality assessment of calcified nodules and systemic inflammatory modulation with PCSK9 inhibition.** This figure summarizes the intracoronary imaging findings and peripheral blood mononuclear cell (PBMC) transcriptomic changes in patients with calcified nodules and stable coronary artery disease treated with Evolocumab on background statin therapy. Serial OCT and NIRS–IVUS imaging demonstrate a significant reduction in maxLCBI_4mmCN_ with a numerical increase in the minimum fibrous cap thickness at the peak convexity of CN over 26 weeks. Parallel PBMC transcriptomic analysis reveals a reduction in neutrophil-driven inflammatory signalling at follow-up, suggesting systemic attenuation of neutrophil activity accompanying plaque modification.

## Introduction

Atherosclerotic coronary artery disease (CAD) remains the leading cause of morbidity and mortality in the USA.^1^ Evolocumab, a potent lipid-lowering agent and proprotein convertase subtilisin/kexin type 9 (PCSK9) inhibitor, increases hepatic low-density lipoprotein cholesterol (LDL-C) receptor expression, leading to substantial reductions in circulating LDL-C, slowing the progression of coronary atherosclerosis and producing incremental improvements in cardiovascular outcomes.^2–5^ Calcified plaques have traditionally been regarded as advanced, relatively quiescent atheroma. However, pathological studies have demonstrated the presence of lipid components within calcified lesions, a phenomenon we describe as ‘butter under the rock’ indicating that these plaques may retain biologically active lipid-rich elements. Calcified nodules (CNs) represent a high-risk lesion subset predisposing to thrombotic acute coronary syndrome (ACS), being present in up to 30% of culprit plaques in severely calcified ACS lesions.^6–8^ Optical coherence tomography (OCT) is the only imaging modality capable of visualizing CN features consistent with histopathology. Although intensive lipid-lowering therapy reduces overall plaque burden and cardiovascular events, its specific effects on CNs remain uncertain and are an important focus of ongoing investigation. Conventional intravascular imaging modalities, including OCT, grayscale intravascular ultrasound (IVUS), and computed tomography angiography (CCTA), have limitations in fully characterizing the internal composition of CNs, hindering definitive assessment of therapeutic response.^6^ In contrast, near-infrared spectroscopy–IVUS (NIRS–IVUS) can uniquely identify and quantify lipid-rich necrotic cores within atherosclerotic plaques, including those embedded in CNs.^9^ This enhanced capability provides a critical tool to document potential reductions in lipid burden within CNs, offering insight into whether lipid-lowering therapy can induce favourable morphological changes or regression in these high-risk lesions. The short-term impact of maximal lipid-lowering therapy on the atheroprogression of calcified nodules (CNs) remains unexplored. This study aimed to use serial OCT and NIRS–IVUS to assess morphological changes in CNs over 6 months of intensive lipid-lowering therapy. We hypothesized that the reduction of peri-calcific lipid-rich regions would indicate enhanced plaque stabilization that could lower the risk of calcium-related plaque events. In addition to serial intravascular imaging of CN, we evaluated the transcriptomic characteristics of peripheral blood mononuclear cells (PBMC) isolated from patients presenting with CN before and after Evolocumab therapy.

## Methods

**Figure 1:**
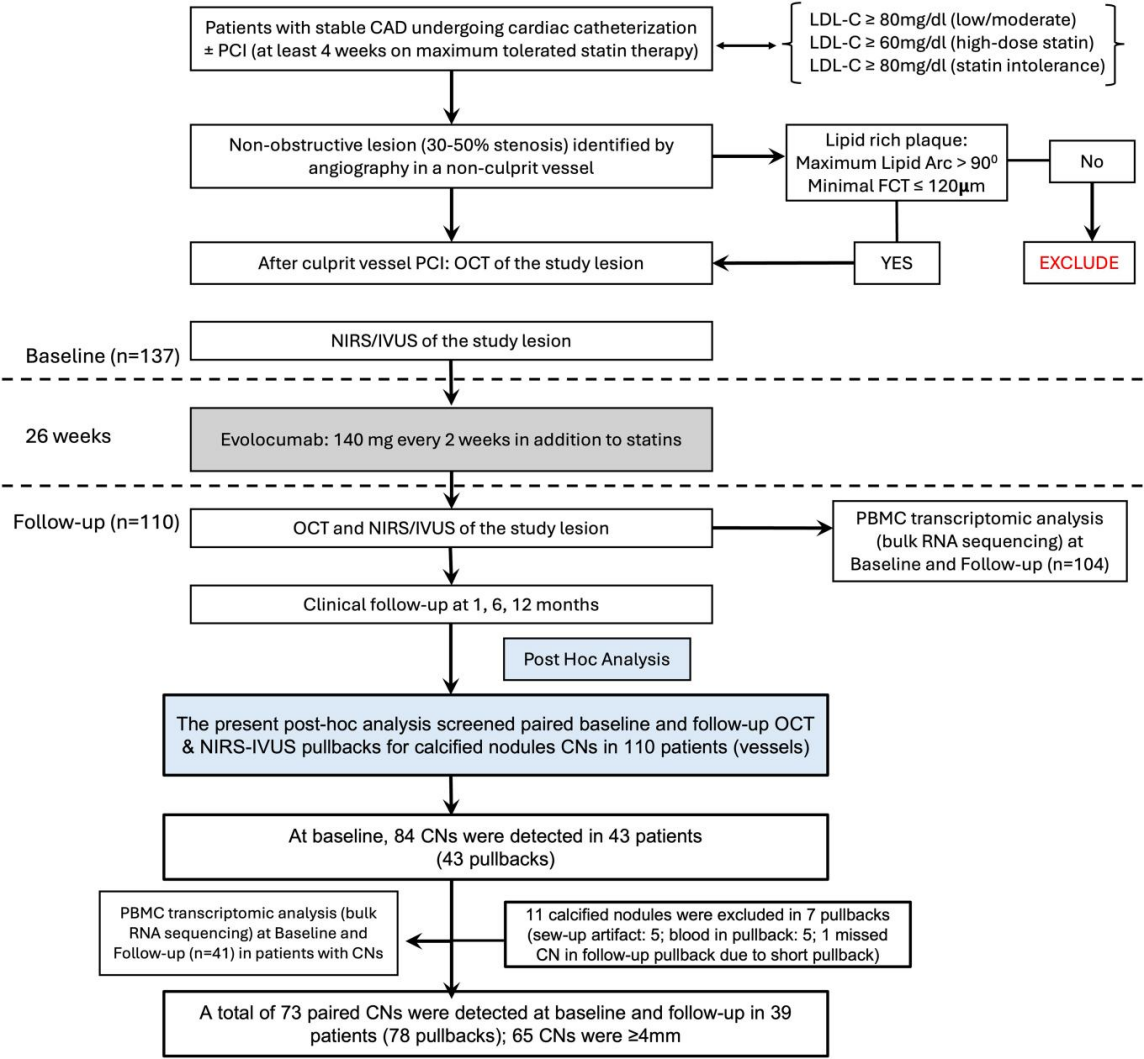
**Study flow chart showing** post-hoc analysis of calcified nodules in YELLOW III. This schematic illustrates the post-hoc selection and analysis of calcified nodules (CNs) from the YELLOW III study. Briefly, YELLOW III enrolled patients with stable coronary artery disease and lipid-rich plaques who underwent baseline OCT and NIRS–IVUS imaging, followed by 26 weeks of Evolocumab therapy on background statin treatment and repeat imaging. For the present post hoc analysis, paired baseline and follow-up OCT and NIRS–IVUS pullbacks from 110 vessels were screened. A total of 84 CNs were identified at baseline in 43 patients. Eleven CNs were excluded because of imaging artefacts or incomplete follow-up pullbacks, resulting in 73 paired CNs in 39 patients for final analysis, including 65 CNs measuring ≥4 mm.

This study included 110 patients presenting with stable CAD and lipid-rich plaque (lipid arc >90^0^) and a minimum fibrous cap thickness (FCT) < 120μm on OCT in a non-obstructive lesion. All patients on maximally tolerated statin therapy received Evolocumab 140 mg biweekly. Lesions were assessed at baseline and 26-week follow-up via OCT, NIRS, and IVUS. The primary imaging endpoint of the YELLOW III study was plaque stabilization, defined as changes in minimum FCT on OCT and maxLCBI4 mm on NIRS. For the present study, CNs were identified by OCT and classified as CNs with or without disruption of the superficial intimal fibrous layer. In the YELLOW III study, after 26 weeks of Evolocumab, OCT fibrous cap thickness (FCT) increased from 70.9 ± 21.7 µm to 97.7 ± 31.1 µm (mean change, 26.8 ± 22.3 µm, *P* < 0.001), and maxLCBI4 mm (LCBI) decreased from 306.8 ± 177.6 to 213.1 ± 168.0 (mean change, −93.7 ± 140.5, *P* < 0.001)^10^. In this post-hoc analysis, 110 patients with paired baseline and follow-up OCT pullbacks were screened for calcified nodules (CNs) and co-localized on NIRS–IVUS pullbacks. At baseline, 84 CNs were identified in 43 patients. After excluding seven follow-up OCT pullbacks due to image quality issues (*Figure 1*), 73 CNs were evaluable in 39 patients. 65 CNs were ≥4 mm. The final analysis, therefore, included 73 CNs with paired baseline and follow-up assessments using OCT and 65 CNs using both OCT and NIRS–IVUS.

### Image acquisition

OCT imaging of the vessel with a non-obstructive lesion detected by coronary angiography was performed using a commercially available frequency-domain OCT system (ILUMIEN™ OPTIS™ System, Abbott Vascular, Santa Clara, CA). Anticoagulation and intracoronary nitroglycerin were administered before imaging. If the OCT inclusion criteria (lipid-rich plaque defined by OCT as a lesion containing at least one image with an FCT ≤120 µm and one image with a lipid arc >90°) were satisfied, a combined NIRS/IVUS pullback was performed using the Makoto Intravascular Imaging System at 0.5 mm/s. During the pullback, grayscale IVUS images were recorded and a NIRS chemogram generated. De-identified OCT and NIRS/IVUS images were analysed at an independent core laboratory (Mount Sinai Hospital Imaging Core Lab) according to prespecified methods described below.

### OCT image analysis

OCT images were analysed using the AptiVue™ Offline Review Software (Abbott Vascular, Santa Clara, CA). Serial OCT images at baseline and 26 weeks of follow-up were reviewed side by side, and the study calcified nodules were identified and matched based on the distance from the anatomical landmarks by a matcher investigator (MK and IB). Calcium was defined as a signal-poor or heterogeneous region with a sharply delineated border.^7^ Calcified nodules were defined according to established OCT criteria as accumulations of small calcium fragments protruding into the lumen, consistent with prior validated reports.^7,11–14^ To enhance diagnostic specificity and reduce potential subjectivity, corresponding IVUS features of convex calcium—defined as intraluminal protrusion >500 μm with a bright echo, irregular/smooth surface, and acoustic shadowing—were also required (PMID: 33221211).^9^ Notably, all calcified nodules identified on OCT in our study fulfilled these IVUS criteria.^9^ The fibrous cap thickness (FCT) over a calcified nodule was measured first at 1-mm intervals and then three times at its thinnest part at each cross-section, and the average value was calculated. In addition, the FCT was also calculated at the peak convexity of CN.^15^ The original and revised calcium scores were calculated as previously described.^16^ The calcium volume index was calculated as calcium length × maximum calcium arc X maximum calcium thickness.^7^ Considering the non-randomized nature of the study, it was impossible to blind core lab personnel to the treatment; therefore, additional measures had been implemented to minimize the potential bias in image analysis. First, all images were analysed by two experienced analysts (YV and PCR) independently. In addition, baseline and follow-up images from the same patient were analysed independently, without side-by-side or sequential review. Instead, baseline pullbacks from several patients were analysed first, followed by the analyses of the corresponding follow-up images.

### NIRS/IVUS image analysis

NIRS chemogram displays the distribution of the probability of lipid-rich plaque (LRP) with the X-axis indicating the pullback position (1 pixel every 0.1 mm) and the Y-axis indicating the circumferential position (1 pixel every 1°). Lipid core burden index (LCBI), the fraction of pixels indicating lipid within a region of interest, was calculated as pixels with a probability of LRP >0.6 divided by all viable pixels multiplied by 1000. The maxLCBI_4mmCN_ was defined as the maximum lipid-core burden index within any 4-mm subsegment of the coronary segment containing a calcified nodule (length ≥4 mm), as identified on co-registered OCT and NIRS–IVUS pullbacks.

### Transcriptomic analysis

Blood samples were collected at baseline and follow-up imaging for PBMC isolation and whole-transcriptome bulk RNA sequencing (RNA-seq) by targeting 50 million reads per sample. Differentially expressed genes (DEG) were identified using DESeq2 with a multi-factor design incorporating patient information to account for inter-sample variability comparing patients with and without CN and adjusting for baseline patient characteristics (sex, age, race, diabetes, hypertension, study lesion, prior CABG). Ingenuity Pathway Analyses (IPA) software (Qiagen Inc., CA) was used to identify enriched molecular pathways. Activation or inhibition of canonical pathways was predicted using an activation *z*-score, with *z* ≥ 2 indicating activation and *z* < *−*2 indicating inhibition.

### Exploratory endpoints

The exploratory endpoint was the change in maxLCBI4 mm of coronary segments harbouring calcified nodules (maxLCBI_4mmCN_) and the change in the morphological features of the calcified nodules, including arc, depth, and fibrous cap thickness.

## Statistical analysis

Categorical data were expressed as numbers and percentages, whereas continuous variables were expressed as mean and (±) SD or as median accompanied by interquartile range as appropriate. Tests of normality were performed using the Shapiro–Wilk test. Continuous variables were compared with Student *t* or Mann–Whitney *U* tests, and categorical variables with chi-square or Fisher's exact tests, as appropriate. Univariable binary logistic regression was used to identify predictors of calcified nodules. Significant correlates (*P* < 0.20) identified on univariable regression were input into the multivariable binary logistic regression model to identify the independent correlates of calcified nodules. Longitudinal changes in imaging endpoints from baseline to 6-month follow-up were analysed using linear mixed-effects models (LMM). To account for the hierarchical nature of the data (multiple nodules nested within individual patients), Patient ID was included as a random intercept. This approach adjusts for within-patient correlation and ensures robust statistical inference. Timepoint was treated as a fixed effect. Covariance structures were evaluated using unstructured and compound symmetry specifications, with the latter providing the most stable fit for the two-timepoint design. To address multiple comparisons in transcriptomics, differential expression analyses were adjusted using the Benjamini–Hochberg false discovery rate (FDR) method, with an adjusted *P*-value (*q*-value) threshold of <0.05 considered statistically significant. For the interobserver variability of quantitative OCT parameters, the degree of agreement between observers was quantified using the intraclass correlation coefficient (ICC). A two-way random-effects model (ICC 2,1) was employed to assess absolute agreement between single measurements. ICC values were interpreted as follows: <0.50 (poor), 0.50–0.75 (moderate),0.75–0.90 (good), and >0.90 (excellent) reliability. In addition to ICC, Bland-Altman analysis was used to visualize the agreement and identify potential systematic bias (see Supplementary data online, *Table S1* and Supplementary data online, *Figure S1*). The mean difference (bias) and the 95% limits of agreement (defined as the mean difference ±1.96 times the standard deviation of the differences) were calculated and plotted for each parameter. All probability values were 2-sided, and *P* < 0.05 was considered statistically significant. Categorical data are reported as numbers and proportions. All statistical analyses were performed using IBM SPSS Statistics for Windows (version 24.0) and R (version 3.5.2, package: proc, R Foundation for Statistical Computing).

## Results

### Incidence, baseline characteristics, and predictors of calcified nodule

**Figure 2:**
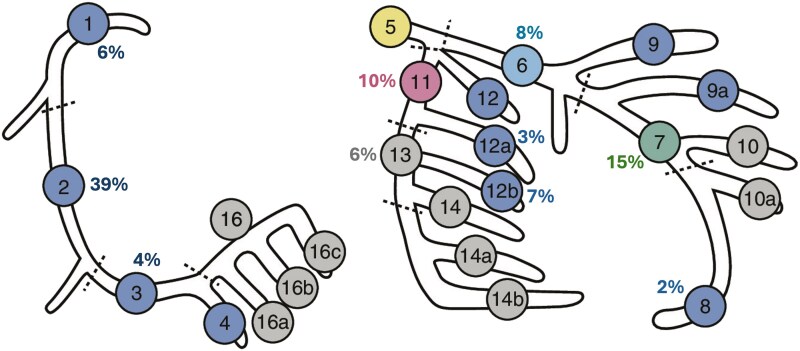
Segmental distribution of calcified nodules assessed by OCT and NIRS–IVUS. Calcified nodules were localized according to standard SYNTAX coronary segmentation. The majority were identified in proximal and mid coronary segments, particularly segment 2 (39%), with additional predilection for segments 7, 11, and 6. Percentages reflect the proportion of total nodules observed in each segment.

In total, 43/110 (39.1%) patients had CNs, most commonly in the RCA (44.2%) and in segment 2 (39%). The segmental distribution of CNs is illustrated in *Figure 2*. In total, 83 CNs were identified at baseline, and 73 CNs were identified at follow-up. Sixty-five CNs were ≥4 mm. Calcified nodules with lipid signals (CN_LS_) on NIRS–IVUS were identified in 43 CNs, and the rest had no lipid signals, which were classified as dense CNs. One CN classified as eruptive at baseline transformed into a non-eruptive phenotype at follow-up, whereas another CN initially classified as non-eruptive at baseline progressed to an eruptive phenotype with overlying thrombus at follow-up. Patients with CNs were older (67.3 ± 9.9 vs 63.2 ± 9.5, *P* =0.035) and more frequently white (79.3% vs 45.2%, *P* = 0.006) as compared with those without CNs (*Table 1*). Patients with CNs were less likely to be hypertensive. Lipid parameters did not differ significantly between the two groups. On univariable analysis, age [Odds Ratio − 1.045 (1.002-1.09), *P* = 0.038], white race [Odds Ratio − 4.63 (1.62-13.22), *P* = 0.004], and hypertension [Odds Ratio − 0.19 (0.036-0.99), *P* = 0.048] were predictors of calcified nodules. However, in multivariable analysis, white race emerged as the only independent predictor of CNs [Odds Ratio − 4.2(1.4-12.4), *P* = 0.010] (*Table 2*).

**Table 1:** Baseline characteristics

	Calcified Nodule (*n* = 43)	No Calcified Nodule (*n* = 67)	*P* value
Age	67.3 (9.9)	63.2 (9.5)	**0**.**035**
BMI	29.7 (26.6–33.5)	29.2 (25.1–31.2)	0.353
Male	35 (81.4%)	46 (68.7%)	0.184
Prior MI	9 (20.9%)	13 (19.4%)	1.000
Prior PCI	32 (74.4%)	45 (67.2%)	0.524
Prior CABG	3 (6.9%)	1 (1.5%)	0.297
Diabetes mellitus	19 (44.2%)	37 (55.2%)	0.329
Hypertension	37 (86%)	65 (97%)	0.054
Current smoking	4 (9.3%)	8 (11.9%)	0.422
Race (*n* = 82)			
White	23 (79.3%)	24 (45.2%)	**0**.**006**
Vessel involved			
LAD	14 (32.6%)	17 (25.4%)	0.515
RCA	19 (44.2%)	24 (35.8%)	0.426
LCX	10 (23.2%)	25 (37.3%)	0.145
RI	0 (0%)	1(1.5%)	1.000
CRP	1.6 (0.8–3.1)	2 (1.05–4.6)	0.220
Total cholesterol	152 (145–181)	159 (140–180)	0.907
LDL	86.6 (70.8–107)	92 (79.2–109.2)	0.489
Triglycerides	112 (79–176)	99 (77–143)	0.328
HDL	44 (36–50)	42 (35–48)	0.354
Follow-up duration (days)	182 (172–189)	182 (171–189)	0.603

BMI—body mass index; CABG—coronary artery bypass grafting; CRP—C reactive protein; MI—myocardial infarction; PCI—percutaneous coronary intervention; HDL—high density lipoprotein; LAD—left anterior descending artery; LDL—low density lipoprotein; LCX—left circumflex artery; RCA—right coronary artery; RI—ramus intermedius.

**Table 2:** Univariable and multivariable predictors of calcium nodule

	Univariable analysis		Multivariable analysis	
	OR (CI)	*P* value	OR (CI)	*P* value
Age	1.045 (1.002–1.09)	**0**.**038**		
BMI	1.03 (0.97–1.1)	0.383		
Male	1.997 (0.79–5.04)	0.143		
Vessel involved				
LAD	1.42 (0.61–3.3)	0.415		
RCA	1.42 (0.65–3.)	0.381		
LCX	0.51 (0.215–1.21)	0.125		
maxLCBI_4mm-CN_	1 (0.998–1.002)	0.98		
CRP	0.97 (0.88–1.065)	0.486		
LDL	0.99 (0.98–1.007)	0.322		
Triglycerides	1 (0.996–1.004)	0.966		
HDL	1.025 (0.985–1.07)	0.230		
Race				
White	4.63 (1.62–13.22)	**0**.**004**	4.2(1.4–12.4)	**0**.**010**
Prior MI	1.1 (0.42–2.85)	0.845		
Prior PCI	1.422 (0.605–3.34)	0.419		
Prior CABG	4.950 (0.49–49.22)	0.172		
Diabetes mellitus	0.642 (0.297–1.39)	0.260		
Hypertension	0.190 (0.036–0.99)	**0**.**048**		

BMI—body mass index; CABG—coronary artery bypass grafting; CN—calcium nodule; CRP—C reactive protein; MI—myocardial infarction; PCI—percutaneous coronary intervention; HDL—high density lipoprotein; LAD—left anterior descending artery; LCBI—Lipid Core Burden Index; LDL—low density lipoprotein; LCX—left circumflex artery; OR—odds ratio; RCA—right coronary artery; RI—ramus intermedius.

### NIRS–IVUS findings in coronary segments with calcium nodules

**Figure 3:**
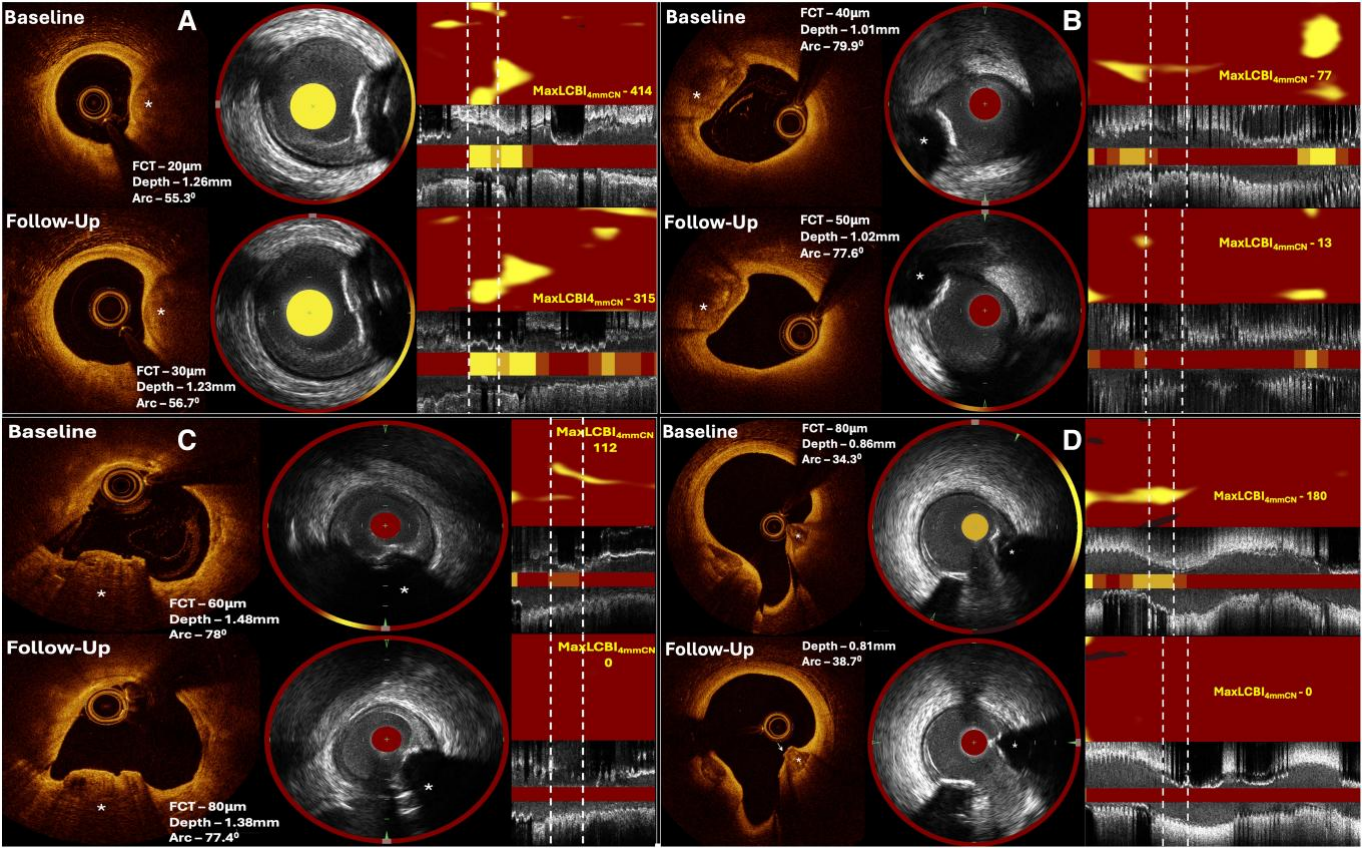
Colocalized OCT and IVUS/NIRS images of calcified nodules before and after maximal medical therapy, showing a reduction in maxLCBI_4mmCN_ at follow-up. The figure illustrates baseline and 6-month follow-up OCT and IVUS–NIRS images of calcified nodules. Four distinct calcified nodules are presented in panels *A*, *B*, *C*, and *D*. All nodules demonstrate a reduction in the lipid core burden index (maxLCBI_4mmCN_) at follow-up. Baseline images demonstrate lipid (“butter”) beneath the calcified nodule (“rock”). Notably, panel *D* depicts a disrupted calcified nodule at 6 months. The figure also highlights morphological changes over time, including variations in fibrous cap thickness (FCT), depth of the calcified nodule at peak convexity, and the maximum calcium arc at the same location.

**Figure 4:**
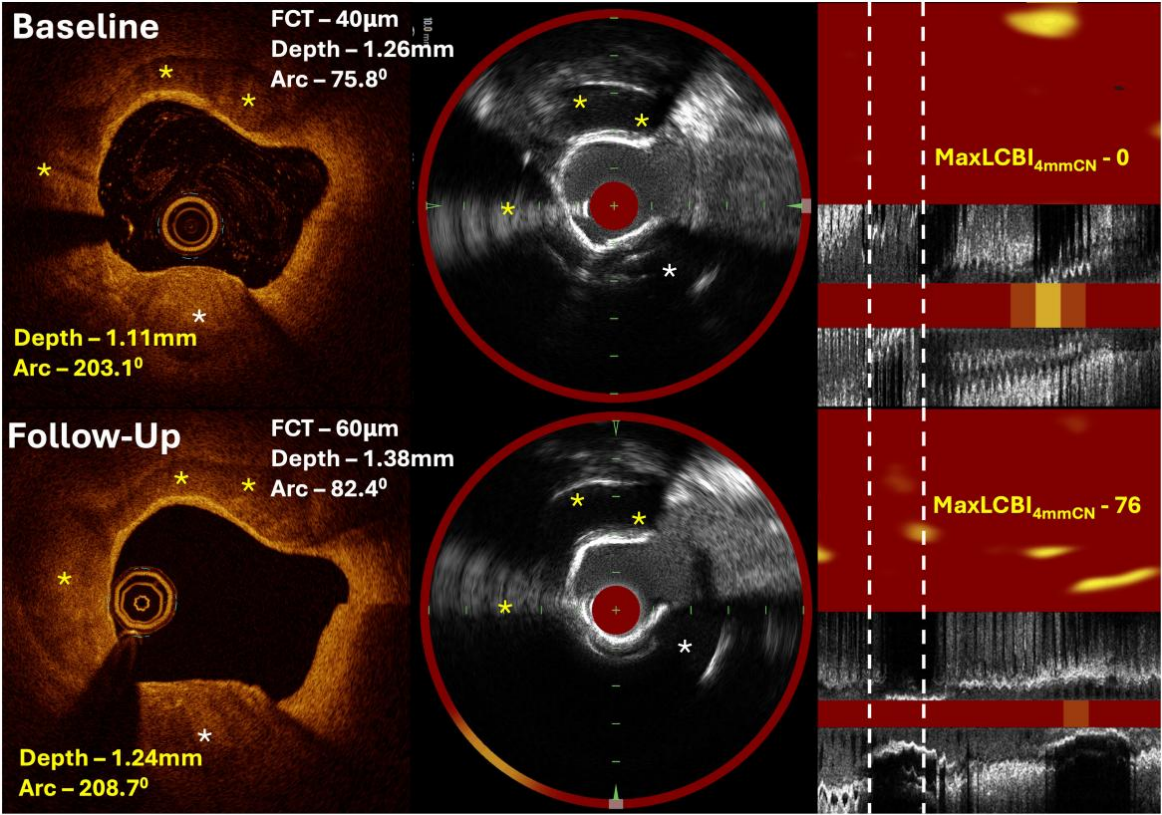
Colocalized OCT and IVUS/NIRS images of calcified nodules before and after maximal medical therapy, showing an increase in maxLCBI_4mmCN_ at follow-up. The figure illustrates baseline and 6-month follow-up OCT and IVUS–NIRS images of a calcified nodule with surrounding calcium. The calcified nodule demonstrates an increase in the lipid core burden index (maxLCBI_4mmCN_) at follow-up. The figure also depicts temporal morphological changes, including alterations in fibrous cap thickness (FCT), the depth of the calcified nodule at peak convexity, and the maximum calcium arc measured at the same site.

The maxLCBI4_mmCN_, decreased significantly from 116.98 ± 123.71 to 75.68 ± 106.46 (Δ −41.31 ± 13.25, *P* = 0.002) (*Table 3*). A reduction in maxLCBI4_mmCN_ was observed in 33 CN-containing segments (50.8%) (*Figure 3*). In 21 segments (32.3%), maxLCBI_4mm-CN_ was 0 at both baseline and follow-up, whereas in 11 segments (16.9%), maxLCBI_4mm-CN_ increased over time (*Figure 4*). A marked and statistically significant reduction in maxLCBI4_mmCN_ was observed in calcified nodules with lipid signal (176.8 ± 111.7 to 113.3 ± 113.7; Δ −63.51 ± 17.63, *P* < 0.001) (*Table 4*), whereas no significant change was seen in dense calcified nodules (Δ + 2.09 ± 2.09, *P* = 0.317) (*Table 4*). Interaction analysis confirmed a significant difference between groups for maxLCBI4_mmCN_ change (*P* = 0.009) (*Table 4*).

**Table 3:** Morphological characteristics of calcium nodule and surrounding calcium before and after maximal lipid-lowering therapy

Variable	Baseline mean	6-Month mean	Mean change (*β*)	Std. error	*P*-value	95% CI
**Calcified nodule (*n*** **=** **73)**						
Arc	97.61 ± 53.13	98.70 ± 53.70	+1.09	7.701	0.888	−14.01, 16.2
Depth	0.93 ± 0.27	0.91 ± 0.24	−0.02	0.034	0.654	−0.08, 0.05
FCT_min_	77.22 ± 71.31	80.14 ± 68.97	+2.92	9.736	0.765	−16.17, 22
Length	5.00 ± 2.57	5.00 ± 2.59	+0.01	0.335	0.987	−0.65, 0.66
Original Ca Score	1.47 ± 0.84	1.50 ± 0.82	+0.03	0.118	0.813	−0.2, 0.26
Revised Ca Score	1.03 ± 0.24	1.03 ± 0.24	0	0.036	1	−0.07, 0.07
CVI	513.43 ± 471.78	511.08 ± 482.79	−2.34	66.732	0.972	−133.1, 128.5
MaxLCBI_4mmCN_ (*n* = 65)	116.98	75.68	−41.31	13.25	**0**.**002**	−67.6, 15.04
**Peak convexity of calcified nodule (*n*** **=** **73)**						
Arc	59.55 ± 19.12	60.24 ± 18.12	+0.69	2.316	0.766	−3.85, 5.23
Depth	0.97 ± 0.29	0.95 ± 0.26	−0.03	0.031	0.389	−0.09, 0.03
FCT_min_	66.53 ± 53.93	75.56 ± 61.93	+9.03	7.802	0.247	−6.3, 24.3
**Surrounding calcium (*n*** **=** **15)**						
Arc	53.10 ± 27.38	54.26 ± 24.53	+1.16	5.151	0.823	−8.9, 11.3
Depth	0.71 ± 0.21	0.71 ± 0.20	+0.00	0.034	0.987	−0.07, 0.07

Ca, calcium; CN, calcium nodule; CVI, Calcium Volume Index; FCT_min,_ minimum fibrous cap thickness; LCBI—Lipid Core Burden Index.

**Table 4:** Morphological characteristics of calcium nodule and surrounding calcium before and after maximal lipid-lowering therapy stratified by lipid signals identified on NIRS–IVUS at baseline

Variable	Calcified nodule with lipid signal (*N* = 43)				Dense calcified nodule (*N* = 22)				
	Baseline	6-Month	Δ Mean (SE)	*P*	Baseline	6-Month	Δ Mean (SE)	*P*	Interaction *P*
**Calcified nodule**									
Arc (°)	100.4 ± 57.7	104.1 ± 61.1	+2.22 (10.99)	0.840	103.0 ± 44.7	105.7 ± 47.2	−0.79 (6.13)	0.897	0.954
Depth (mm)	0.97 ± 0.35	0.92 ± 0.26	−0.03 (0.03)	0.398	0.87 ± 0.22	0.91 ± 0.18	0.02 (0.03)	0.642	0.346
FCT_min_ (µm)	77.0 ± 77.6	82.9 ± 74.7	+3.53 (8.96)	0.693	66.1 ± 64.4	63.2 ± 57.6	−0.22 (17.33)	0.990	0.705
Length (mm)	5.60 ± 2.46	5.63 ± 2.44	+0.03 (0.22)	0.899	6.10 ± 2.41	6.10 ± 2.41	0.00 (0.64)	1.000	0.966
Original Ca score	1.49 ± 0.74	1.58 ± 0.86	+0.09 (0.14)	0.523	1.82 ± 1.01	1.74 ± 0.93	−0.13 (0.17)	0.458	0.541
Revised Ca score	1.05 ± 0.30	1.05 ± 0.32	0.00 (0.06)	0.954	1.00 ± 0.00	1.00 ± 0.00	0.00 (0.00)	1.000	0.931
MaxLCBI_4mmCN_	176.8 ± 111.7	113.3 ± 113.7	**−63.51** (**17.63)**	**<0**.001*	0.00 ± 0.00	2.09 ± 9.81	2.09 (2.09)	0.317	**0**.009*
**Peak convexity**									
FCT_min_ (µm)	64.9 ± 60.8	72.6 ± 71.0	+5.52 (8.74)	0.528	70.9 ± 55.4	71.1 ± 47.4	+3.87 (11.59)	0.738	0.721
Depth (mm)	1.00 ± 0.33	0.97 ± 0.27	−0.02 (0.02)	0.358	0.95 ± 0.24	0.97 ± 0.18	0.01 (0.06)	0.853	0.821
Arc (°)	59.1 ± 18.8	60.6 ± 18.4	+1.17 (2.13)	0.582	63.4 ± 18.1	63.1 ± 20.1	−0.66 (3.75)	0.861	0.627
**Surrounding calcium**									
Arc (°)	40.8 ± 13.1	43.9 ± 13.5	+2.78 (1.18)	0.018*	67.8 ± 40.7	65.6 ± 36.5	−2.18 (3.18)	0.493	0.081
Depth (mm)	0.65 ± 0.25	0.69 ± 0.26	+0.04 (0.02)	0.057	0.80 ± 0.11	0.74 ± 0.08	−0.06 (0.04)	0.120	0.012*

Ca—calcium; FCT_min_—minimum fibrous cap thickness; CN, calcium nodule; LCBI, Lipid Core Burden Index; NIRS–IVUS, near infrared spectroscopy–intravascular ultrasound.

### Morphological changes of calcified nodule

**Figure 5:**
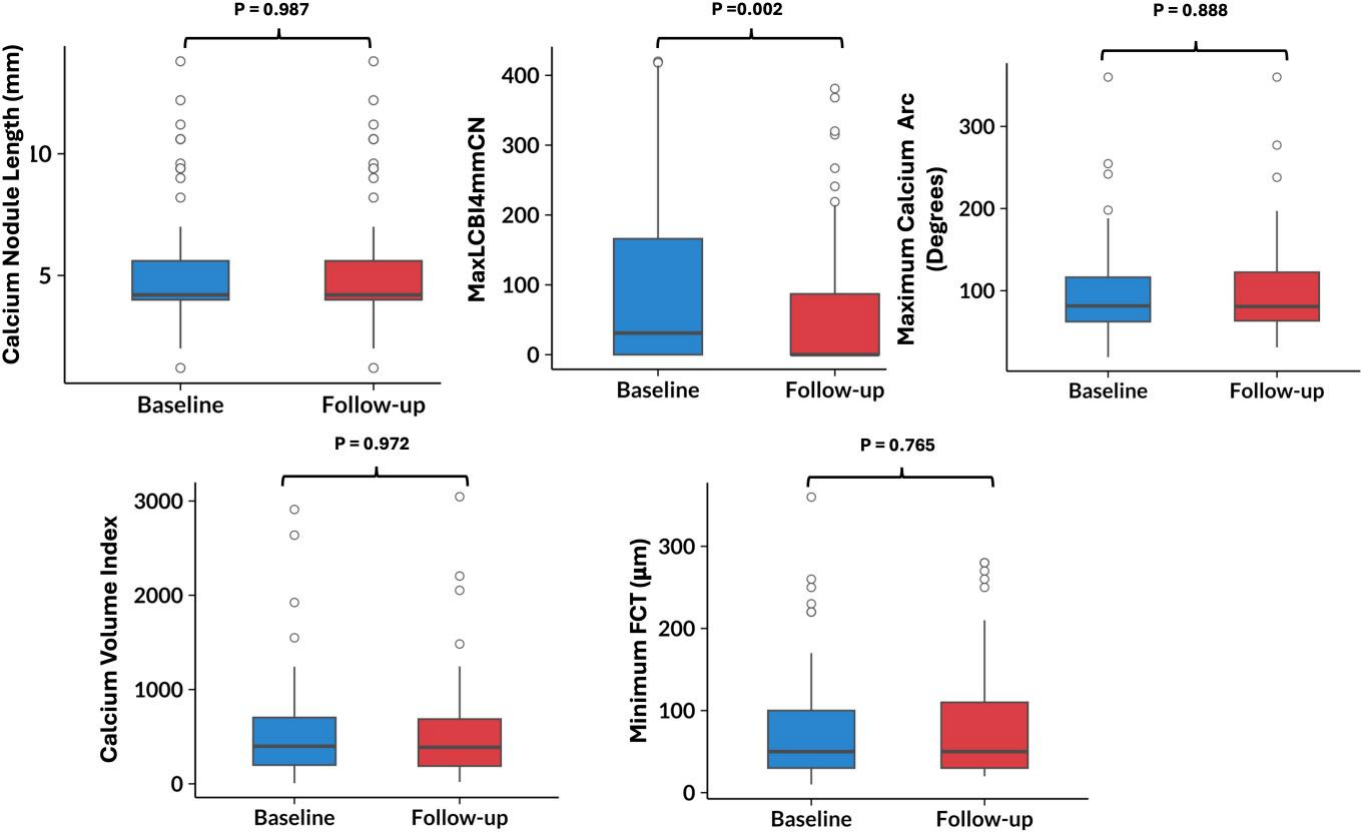
Various morphological measurements of calcified nodules, before and after maximal medical therapy. A significant reduction was observed in calcified nodule length, maximum lipid core burden index over 4 mm (maxLCBI4mmCN), and minimum fibrous cap thickness (FCT) at follow-up. No significant differences were noted in the maximum calcium arc or calcium volume index.

**Calcified nodule:** At 6-month follow-up, there were no significant changes in most morphological characteristics of calcified nodules (*Figure 5*, *Table 3*). Specifically, calcified nodule arc increased slightly from 97.61 to 98.7 (*Δ* + 1.09 ± 7.70, *P* = 0.888), while depth showed a small decrease from 0.93 to 0.91 (*Δ* −0.02 ± 0.03, *P* = 0.654). Fibrous cap thickness (FCT) increased from 77.22 to 80.14 µm (*Δ* + 2.92 ± 9.74, *P* = 0.765), and nodule length remained essentially unchanged (5.0–5.0 mm; *Δ* + 0.01 ± 0.34, *P* = 0.987). Similarly, original calcium score (1.47–1.50; Δ + 0.03 ± 0.12, *P* = 0.813), revised calcium score (1.03–1.03; Δ 0.00 ± 0.04, *P* = 1.000), and calcium volume index (CVI; 513.43–511.08; *Δ* −2.34 ± 66.73, *P* = 0.972) did not change significantly.

**Peak convexity of the calcified nodule (see Supplementary data online, *Figure S2*):** At the peak convexity of calcified nodules, no significant differences were observed over time. Arc increased from 59.55 to 60.24 (*Δ* + 0.69 ± 2.32, *P* = 0.766), depth decreased slightly from 0.97 to 0.95 (*Δ* −0.03 ± 0.03, *P* = 0.389), and FCT increased from 66.53 to 75.56 µm (*Δ* + 9.03 ± 7.80, *P* = 0.247), though none of these changes reached statistical significance.

**Calcium surrounding the CN:** Similarly, in the surrounding calcium, arc increased from 53.1 to 54.26 (*Δ* + 1.16 ± 5.15, *P* = 0.823), and depth remained unchanged (0.71–0.71; *Δ* + 0.00 ± 0.03, *P* = 0.987), with no statistically significant differences.

### Calcified nodules stratified by lipid signals (***Table 4***)

When stratified by calcified nodules with lipid signal (*N* = 43) vs. dense calcified nodules (*N* = 22), there were no significant changes in arc, depth, FCT, length, original calcium score, revised calcium score, or CVI in either group. No significant longitudinal changes were observed in parameters at the peak convexity in either group. In nodules with lipid signal, FCT increased from 64.9 ± 60.8 to 72.6 ± 71.0 µm (*Δ* + 5.52 ± 8.74, *P* = 0.528), depth decreased from 1.00 ± 0.33 to 0.97 ± 0.27 mm (*Δ* −0.02 ± 0.02, *P* = 0.358), and arc increased from 59.1 ± 18.8 to 60.6 ± 18.4° (*Δ* + 1.17 ± 2.13, *P* = 0.582). Comparable non-significant changes were observed in dense calcified nodules (all *P* > 0.7), with no significant interaction between groups.

In the calcium surrounding CNs, nodules with lipid signal demonstrated a modest but statistically significant increase in arc (40.8 ± 13.1 to 43.9 ± 13.5°; *Δ* + 2.78 ± 1.18, *P* = 0.018), whereas depth showed a trend toward increase (0.65 ± 0.25 to 0.69 ± 0.26 mm; *Δ* + 0.04 ± 0.02, *P* = 0.057). In contrast, dense calcified nodules showed no significant changes in surrounding calcium arc (*Δ* −2.18 ± 3.18, *P* = 0.493) or depth (*Δ* −0.06 ± 0.04, *P* = 0.120). Interaction testing revealed a significant difference between groups for surrounding calcium depth (*p*_interaction_ = 0.012), but not for arc (*p*_interaction_ = 0.081).

### Non-eruptive vs. eruptive calcified nodules

Among 73 calcified nodules (CNs) identified at baseline, one (1.4%) eruptive CN transformed into a non-eruptive nodule at follow-up (see Supplementary data online, *Figure S3*), suggesting healing of the disrupted surface. Conversely, one baseline non-eruptive CN progressed to an eruptive phenotype at follow-up (see Supplementary data online, *Figure S4*). Because only a single eruptive calcified nodule was identified at baseline, subgroup analyses comparing eruptive vs. non-eruptive lesions were not performed.

**Figure 6:**
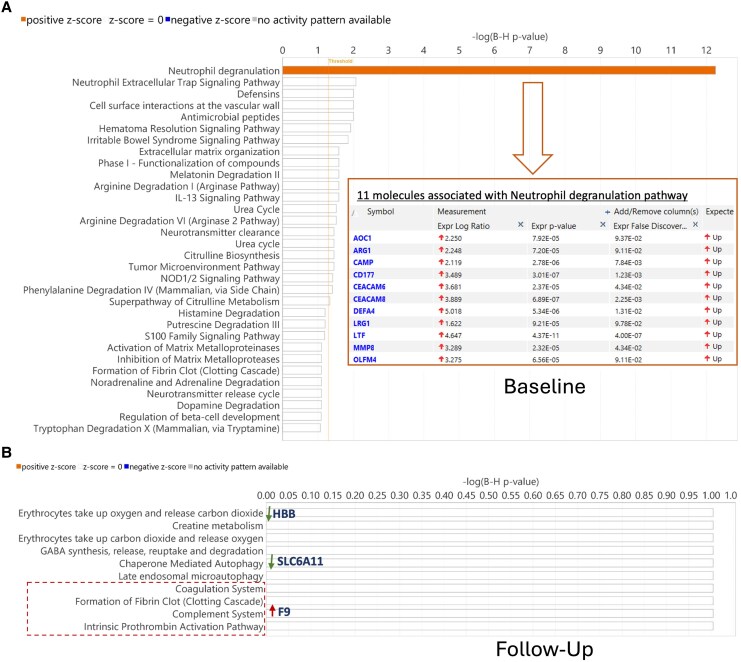
Transcriptomic analysis of PBMCs isolated from patients with and without CN at baseline and follow-up. *(A*) ‘Neutrophil degranulation’ canonical pathway was activated in patients with CN before evolocumab therapy (z-score 3.3). *(B*) Significant canonical pathways in patients with CN after evolocumab therapy and key genes (green arrows indicate downregulation, red arrow upregulation).

**Transcriptomic Analysis (*Figure 6*).**

A total of 110 patients completed the 6-month follow-up in the YELLOW III study. Transcriptomic data at both baseline and follow-up were available for 104 patients. Importantly, transcriptomic data were available for 41 (95.3%) of 43 patients with calcified nodules included in the present analysis (*Figure 1*). The DEG profile of PBMC at baseline identified 35 transcripts, 13 up-regulated and 22 down-regulated genes, differentially expressed in patients with CN compared to patients without CN (see Supplementary data online, *Table S1*). Out of the 13 up-regulated genes, 11 were associated with an activated canonical pathway regulating ‘Neutrophil Degranulation’, z-score =3.3 (orange bar and insert in *Figure 6A*). In addition, the presence of CN was significantly associated with the ‘Neutrophil Extracellular Trap (NET) Signalling’ pathway, with 4 key genes demonstrating substantial upregulation in PBMC from CN patients (CAMP, DEFA3, DEFA4, LTF) (see Supplementary data online, *Table S1*). DEG analysis at follow-up identified 57 transcripts differently expressed in CN patients (see Supplementary data online, *Table S2*), however, none of the canonical pathways reached the threshold for significance and activation/inhibition (*Figure 6B*). In contrast to baseline analysis, the expression levels of genes associated with neutrophil degranulation and NET formation were similar in patients with and without CN after Evolocumab therapy. Increased levels of F9 gene expression (coagulation Factor IX) in the CN group were associated with several prothrombotic pathways (red rectangular in *Figure 6B*), remaining significant after Evolocumab therapy.

## Discussion

This serial OCT and NIRS/IVUS investigation is the first to report the atheroprogression/regression of pre-existing CN at short term in patients receiving maximal lipid-lowering therapy. The main findings of this study can be summarized as follows: (i) First, lipid signal was detected in 66.2% of coronary segments harbouring calcified nodules on NIRS–IVUS; these were classified as calcified nodules with lipid signal (CN_LS_), whereas the remaining lesions without detectable lipid were categorized as dense calcified nodules (ii) Treatment with intensive lipid-lowering therapy, consisting of high-dose statins in combination with Evolocumab, was associated with a significant reduction in maxLCBI4_mmCN_ within calcified nodules exhibiting lipid signal on NIRS–IVUS at baseline.(iii) Despite aggressive lipid lowering, the overall morphological characteristics of calcified nodules remained largely stable in both groups, although a numerical increase in fibrous cap thickness at the site of peak convexity was observed. (iv) In CNL_S_ there was a significant increase in calcium arc of the surrounding calcium, accompanied by a trend toward increased depth in surrounding calcium (v) PBMC transcriptomic analysis demonstrated that, in patients with calcified nodules receiving maximally tolerated statin therapy, neutrophil degranulation and NET-related pathways were highly enriched at baseline, whereas at follow-up these inflammatory pathways lost significance and coagulation-related pathways predominated, suggesting attenuation of neutrophil activity with PCSK9 inhibition.

Previous studies have reported that the incidence of new CNs tends to increase beyond two years after baseline OCT.^7^ In our study, no new CNs emerged, nor did existing CNs disappear, likely reflecting the short-term duration of follow-up. Consistent with prior observations, CNs were more frequently detected in older patients, in individuals of White race, and in the right coronary artery.^6–8^ Nearly all CNs identified at both baseline and follow-up were non-eruptive, with only one exception at each time point, consistent with the stable CAD presentation of the study cohort. The incidence of calcified nodules (CNs) in our cohort was relatively high (39.1%). A comparable prevalence was reported in the PROSPECT study, which identified non-culprit CNs in approximately 30% of patients using three-vessel grayscale IVUS and IVUS–virtual histology, with most being clinically benign.^17^ Our institution serves as a referral centre for high-risk percutaneous coronary intervention in patients with complex and heavily calcified coronary artery disease, which may have contributed to a selection bias and the higher observed prevalence of CN. In addition, the higher incidence observed in our study likely reflects the superior spatial resolution of optical coherence tomography (OCT) compared with intravascular ultrasound. Unlike IVUS, which lacks sufficient resolution to detect small nodular calcifications reliably, OCT enables high-resolution visualization of coronary calcium and CN morphology, facilitating the identification of even subtle or early-stage lesions.

Near-infrared spectroscopy (NIRS) detects lipid-rich plaque based on spectroscopic signal rather than direct quantification of lipid content. Prior histopathological validation studies have demonstrated high specificity of NIRS for identifying lipid-rich plaques, supporting its reliability in intracoronary imaging. Importantly, available evidence suggests that the presence of calcium does not substantially impair the detection of lipid signal by NIRS, and lipid-rich plaques can be identified even within calcified lesions. In a large acute myocardial infarction cohort (*n* = 244) using optical coherence tomography as the reference standard, IVUS-defined convex calcium identified calcified nodules with high sensitivity (93%) and specificity (100%). NIRS-derived maxLCBI4 mm values were consistently measurable in calcified nodules (butter under the rock) and were intermediate between plaque rupture (median 705 [IQR: 545–854]) and plaque erosion (median 300 [IQR: 126–357]), with calcified nodules demonstrating median values of 355 (IQR: 303–478; *P* < 0.001). A combined NIRS–IVUS algorithm incorporating plaque cavity, convex calcium, and maxLCBI4 mm achieved excellent diagnostic performance for calcified nodules, with a sensitivity of 100% and specificity of 99% in the validation cohort. Complementary evidence from the REASSURE-NIRS registry, including 65 calcified lesions (325 cross-sectional frames), demonstrated that the NIRS-detected lipid signal was present in 84.3% of calcified frames. A greater lipid signal (yellow signal arc ≥63°) was independently associated with thinner calcification (*β* = −0.446, *P* < 0.001), further supporting the biological plausibility of lipid detection within calcified plaques. Collectively, these data indicate that NIRS can reliably detect lipid signal in calcified coronary lesions, supporting its use in compositional plaque assessment despite the presence of calcium. Nevertheless, NIRS measurements may still be influenced by signal attenuation, imaging artifacts, and changes in catheter position, particularly in complex or heavily calcified segments. Therefore, the observed reduction in maxLCBI_4mmCN_ should be interpreted as a change in NIRS-derived lipid signal rather than definitive evidence of plaque delipidation, and these findings warrant confirmation using complementary imaging modalities or histopathological correlation.

Previous studies suggest that CNs arise predominantly from necrotic core calcification rather than collagen-rich calcification, and our findings appear to support this hypothesis.^7,18^ These results reinforce the concept that CNs harbour residual lipid components that are potentially responsive to lipid-lowering therapy, as observed in our study. In our study, the median baseline maxLCBI_4mmCN_ was substantially lower (100) compared with the 355 reported previously,^9^ consistent with a stable CAD population and a larger sample of CNs (n = 65) vs. only 25 CNs in the ACS cohort of the prior study. Furthermore, prior ex vivo histopathological and OCT studies have demonstrated that calcified plaques with strong attenuation frequently contain residual lipid, either adjacent to or within calcium, with 45% of superficial dense calcium exhibiting an indistinct outer border consistent with residual necrotic core.^12,19^

Several studies have reported that long-term statin use may be associated with progression of coronary artery calcification (CAC) scores,^20,21^ whereas more recent evidence suggests that PCSK9 inhibitors can attenuate CAC progression in patients with CAD.^22^ In our study, we demonstrated a reduction in lipid burden (LCBI) within CN-containing segments (maxLCBI_4mmCN_). If the lipid core is indeed a driver of CN formation and progression, its reduction could theoretically slow CN growth, render morphology less protrusive or rupture-prone, and decrease the likelihood of intraplaque haemorrhage. Although robust direct evidence of CN regression with lipid-lowering therapies is still lacking, the emerging recognition that CNs may harbour lipid-rich cores, together with the established effects of intensive lipid-lowering on overall plaque burden and composition, provides a strong biological rationale for further investigation—particularly in physiologically insignificant CNs.^23^

Given that this study was designed as an exploratory mechanistic imaging substudy, it was not powered or intended to evaluate clinical endpoints. The primary focus was to assess changes in plaque morphology and lipid burden specifically related to calcified nodules using OCT and NIRS–IVUS. While prior large-scale outcome trials, such as the FOURIER and ODYSSEY OUTCOMES, have established the clinical benefit of PCSK9 inhibition, the present study does not establish a direct link between calcified nodule modification and clinical event reduction. Accordingly, these findings should be interpreted as hypothesis-generating and warrant validation in studies incorporating longitudinal clinical outcomes. Therefore, the observed imaging changes should be interpreted within the context of surrogate endpoint modulation rather than definitive clinical benefit. In FOURIER (n = 27 564; median follow-up 2.2 years), Evolocumab achieved a ∼59% reduction in LDL-C (to a median of 30 mg/dL) and significantly reduced the primary composite endpoint (CV death, MI, stroke, unstable angina, or revascularization) (HR 0.85, 95% CI 0.79–0.92; *P* < 0.001), with a 20% relative reduction in the key secondary endpoint (CV death, MI, or stroke) (HR 0.80, 95% CI 0.73–0.88; *P* < 0.001). Similarly, in ODYSSEY OUTCOMES (n = 18 924; median follow-up 2.8 years), alirocumab reduced the risk of recurrent ischaemic events after ACS (primary endpoint HR 0.85, 95% CI 0.78–0.93; *P* < 0.001), with a signal towards reduced all-cause mortality (HR 0.85, 95% CI 0.73–0.98). Taken together, these trials provide robust clinical evidence that aggressive LDL-C lowering improves hard cardiovascular outcomes. Therefore, while the mechanistic imaging changes observed in the current study are hypothesis-generating and cannot be directly extrapolated to clinical benefit, they are biologically plausible and directionally consistent with outcome data from these landmark trials.

The presence of OCT-defined CN was associated with up-regulated neutrophil degranulation signalling in patient PBMC at baseline, suggesting a potential role of neutrophils in CN formation. Significant differences in baseline transcriptome have been observed despite all patients presenting with stable CAD and treated with high-intensity statins at baseline. There were no significant differences in neutrophil-related genes after 26 weeks of Evolocumab. Neutrophils are a vital component of the innate immune system. As part of their antimicrobial functions, activated neutrophils can release enzymes from granules (degranulation) and form neutrophil extracellular traps (NETs), extracellular web-like structures. Excessive NETs can contribute to the onset and progression of CVDs by exacerbating inflammatory responses and promoting thrombosis.^24^ NETs play a central role in thrombosis by promoting fibrin deposition and formation of fibrin.^25^ During acute MI, neutrophils interact with platelets at the site of plaque rupture, further increasing the thrombogenic potential of NETs. Key components of NETs, Nucleosomes (DNA-histone complexes), and double-stranded DNA (dsDNA) have been detected at increased levels at the culprit site and correlate with the thrombus burden and infarct size. NETs trapped within the fibrin strands might have a role in plaque erosion through an increase in endoplasmic reticulum (ER), inducing ER stress and apoptosis accompanied by Toll-like receptor (TLR)-2 stimulation.^26^ Targeted nanoparticles delivering an agent that limits NETs formation might represent a novel therapeutic approach to plaque erosion.^27^

After 26 weeks of Evolocumab, several genes implicated in atherosclerosis progression and atherothrombosis were associated with the presence of CN (see Supplementary data online, *Table S2*). A recently discovered circulating lncRNA, atherosclerotic plaque pathogenesis associated transcript (APPAT), had reduced expression in coronary artery samples with severe stenosis and cholesterol treated smooth muscle cells.^28^ Elevated levels of F9 (coagulation Factor IX), a key component of coagulation cascade, have been shown to contribute to clot formation after plaque rupture.^29^ CCL21 (C-C motif chemokine ligand 21) is abnormally elevated in coronary artery disease, can enhance platelet activation and exacerbates thrombus formation.^30^

At baseline, transcriptomic pathway analysis demonstrated strong enrichment of neutrophil degranulation and NET-related signalling, with up-regulation of multiple neutrophil effector molecules, whereas at follow-up these inflammatory pathways lost significance and the dominant residual signal shifted towards coagulation and fibrin-clot-related pathways. This suggests that lipid lowering—potentially enhanced by PCSK9 inhibition—may attenuate neutrophil-driven inflammatory mechanisms, but does not fully mitigate the persistent pro-thrombotic milieu. Further studies are warranted to better understand the underlying molecular mechanisms and the real impact of NETs on CN progression in the patient population to develop potential therapeutic strategies. Given that transcriptomic profiling was performed on peripheral blood mononuclear cells (PBMCs), the findings reflect systemic immune signalling rather than plaque-specific biology. While the observed shift from neutrophil degranulation pathways towards coagulation-related signalling is biologically plausible, no direct causal relationship between these systemic changes and local plaque stabilization can be inferred. Furthermore, the relatively small sample size and high-dimensional nature of transcriptomic data introduce the potential for residual confounding and false-positive findings despite statistical correction. Therefore, these results should be interpreted as associative and exploratory, providing insights into systemic biological responses to intensive lipid-lowering therapy rather than definitive mechanistic evidence.

Further research leveraging high-resolution intravascular and non-invasive imaging is essential to clarify the precise impact of aggressive lipid-lowering on this distinct and increasingly recognized culprit lesion in ACS. Longitudinal studies with larger cohorts and extended follow-up will be critical to determine whether modifying the lipid content of CNs can translate into durable morphological stabilization and, ultimately, improved clinical outcomes.

### Limitations

First, our study was conducted at a single centre with a relatively small sample size, which may limit the generalizability of the findings. Second, the observational design precludes any inference of causality between changes in lipid burden and CN characteristics. Third, our study did not include systematic three-vessel OCT evaluation and therefore may have missed calcified nodules in non-imaged vessels. However, the non-intervened vessels (PCI or imaging) in our cohort were angiographically disease-free and thus were not considered for OCT imaging or PCI. Fourth, we acknowledge as a key limitation the limited representation of eruptive calcified nodules in our cohort, which restricts the generalizability of our findings across all calcified nodule phenotypes. Accordingly, our results should be interpreted with caution and not extrapolated to all CN subtypes. Larger studies with more balanced representation, particularly including a greater number of eruptive CNs, are needed to better delineate potential differences in plaque evolution and response to intensive lipid-lowering therapy across distinct CN phenotypes. Fifth, the analysis was restricted to non-obstructive lesions in patients with stable coronary artery disease, which may not reflect the biological and morphological characteristics observed in acute coronary syndrome settings. Sixth, cases with suboptimal optical coherence tomography (OCT) image quality were excluded, potentially introducing selection bias towards more favourable imaging conditions and lesion characteristics. Seventh, heavily calcified obstructive lesions and complex real-world percutaneous coronary intervention (PCI) scenarios were not represented in this cohort. As such, the generalizability of our findings to ACS populations, advanced calcific disease, and routine clinical practice may be limited. These results should therefore be considered hypothesis-generating and warrant validation in broader, unselected populations. Eighth, given the 26-week duration of follow-up, the study captures short-term changes in plaque composition rather than the long-term evolution of calcified atherosclerosis, which typically occurs over years. While significant reductions in lipid burden, these findings should not be interpreted as evidence of regression of calcified plaque. In particular, structural features such as the calcium arc are unlikely to undergo meaningful modification over this timeframe. Accordingly, the results reflect early stabilization signals rather than definitive structural remodelling, and longer-term studies are required to assess the durability and clinical relevance of these changes. Nineth, formal power calculations were not performed due to the exploratory nature of the transcriptomic analysis of calcified nodules; therefore, the results should be interpreted as hypothesis-generating, mechanistic study and warrant validation in larger, independent cohorts.

## Supplementary Material

qyag092_Supplementary_Data

## Data Availability

The data underlying this article will be shared on reasonable request to the corresponding author.

## Lead author biography

Dr. Pruthvi C. Revaiah completed his residency training in Internal Medicine and Cardiology at the prestigious Postgraduate Institute of Medical Education and Research (PGIMER), Chandigarh, India, in 2020. He subsequently earned his PhD at the CORRIB Research Centre for Advanced Imaging and Core Laboratory at the University of Galway, Ireland. He is currently an Interventional Cardiology Fellow at Mount Sinai Hospital in New York, United States of America. Following his fellowship in complex coronary interventions, he aims to pursue training in structural heart interventions and continue his research in coronary artery disease and structural heart disease, to contribute meaningfully to the literature.
