# Supplementary material for: Intra-coronary imaging and transcriptomics of calcified nodules before and after intensive lipid-lowering therapy: a YELLOW III substudy
Source: Eur Heart J Imaging Methods Pract. 2026 Jun 19;4(1):qyag092. doi: 10.1093/ehjimp/qyag092 (PMC13282941; doi:10.1093/ehjimp/qyag092)
Supplement: qyag092_Supplementary_Data [file qyag092_supplementary_data.docx]

**Intra-Coronary Imaging and Transcriptomics of Calcified Nodules Before And After Intensive Lipid-Lowering Therapy: A YELLOW III Substudy**

**Supplementary Figure 1. Bland Altman Analysis for interobserver variability of quantitative OCT measurements.** Interobserver reproducibility between two independent observers (Observer 1 and Observer 2) was evaluated for three primary categories of measurements: (1) Calcified Nodule (CN) parameters, including minimum fibrous cap thickness (FCT), calcium arc, calcium depth, and calcium length; (2) Surrounding Calcium parameters, including calcium arc and depth; and (3) measurements at the Peak Convexity of the CN, including calcium arc, minimum FCT, and calcium depth. Bland-Altman plots confirmed high levels of agreement with negligible systematic bias across the range of measurements for fibrous cap thickness, calcium arc, and depth


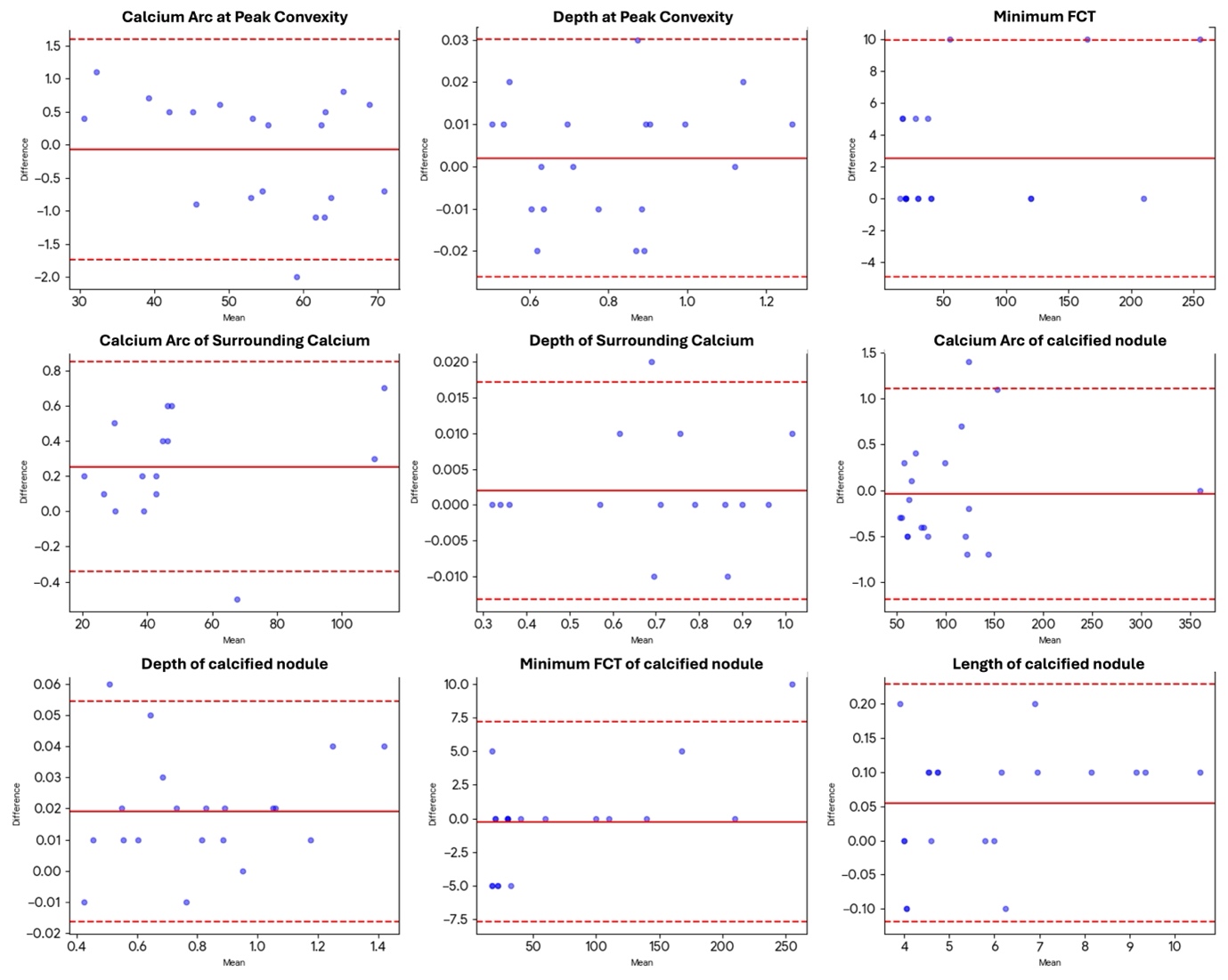


**Supplementary Figure 2. Various morphological measurements of the calcified nodule at the peak convexity**

A significant reduction was observed in the minimum FCT and calcified nodule depth. No significant difference was noted in the maximum calcium arc.

**
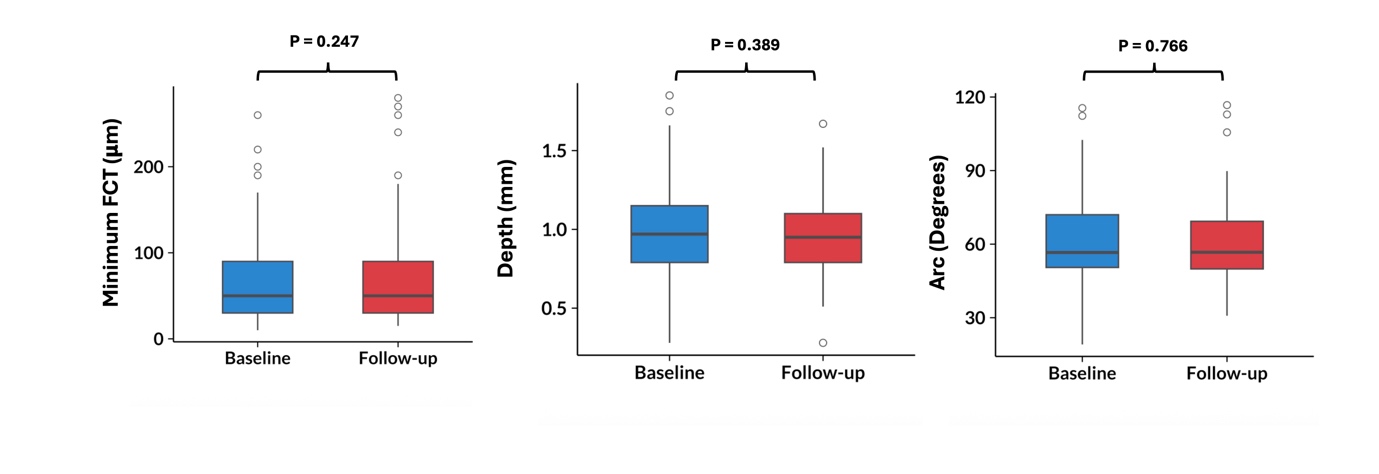
**

**Supplementary Figure 3. OCT images showing a healed eruptive calcified nodule at follow-up**

The figure illustrates baseline and six-month follow-up OCT and IVUS-NIRS images of a calcified nodule at 2 consecutive frames. The eruptive calcified nodule (irregular surface with attached thrombus) at baseline seems to heal at follow-up.

**
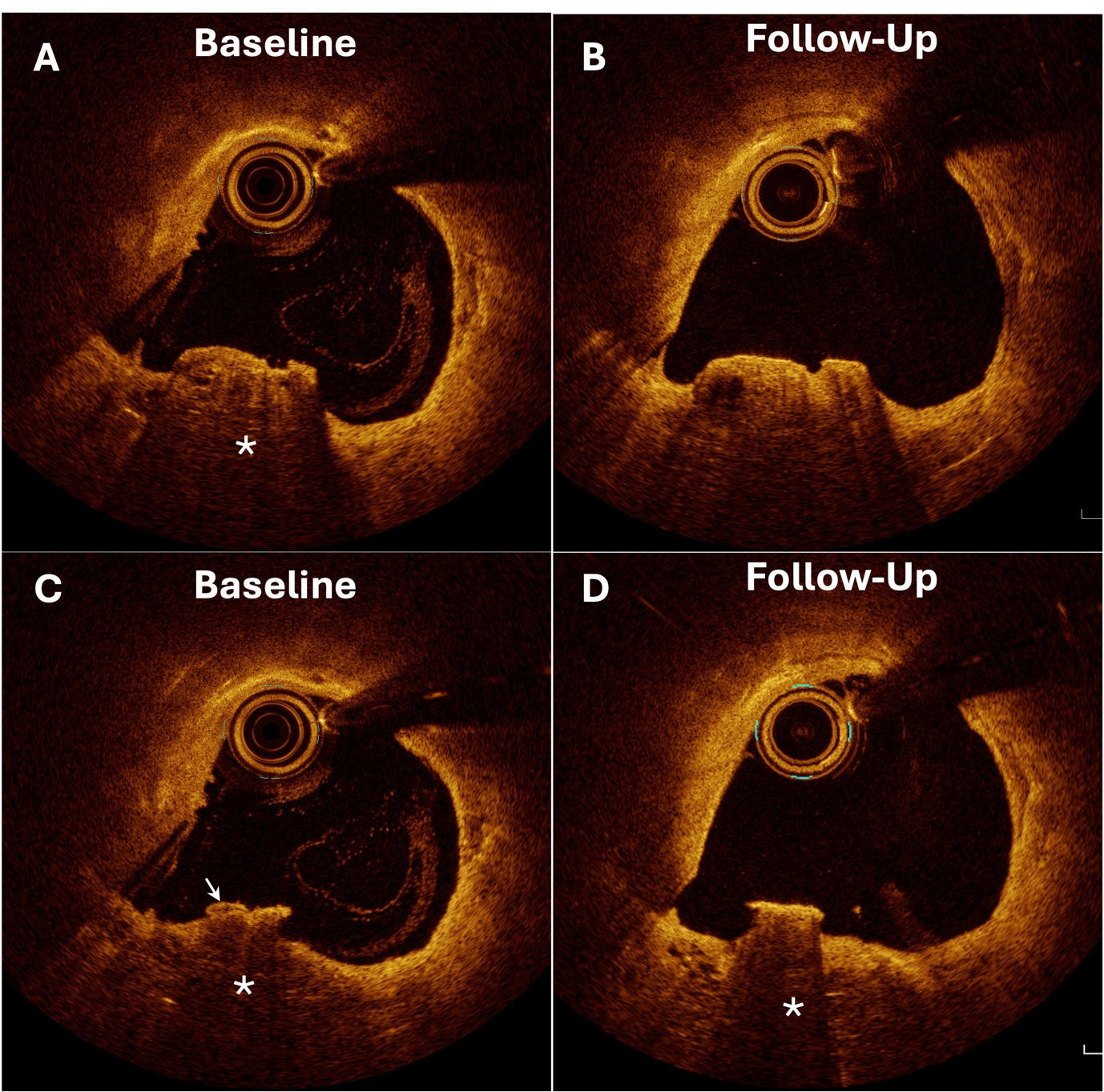
**

**Supplementary Figure 4. Evolution of a non-eruptive calcified nodule into an eruptive calcified nodule, indicating plaque surface disruption**

The figure illustrates baseline and six-month follow-up OCT and IVUS-NIRS images of a calcified nodule at two consecutive frames. The previously non-eruptive calcified nodule at baseline appears disrupted at follow-up, characterized by an irregular luminal surface and a small adherent thrombus


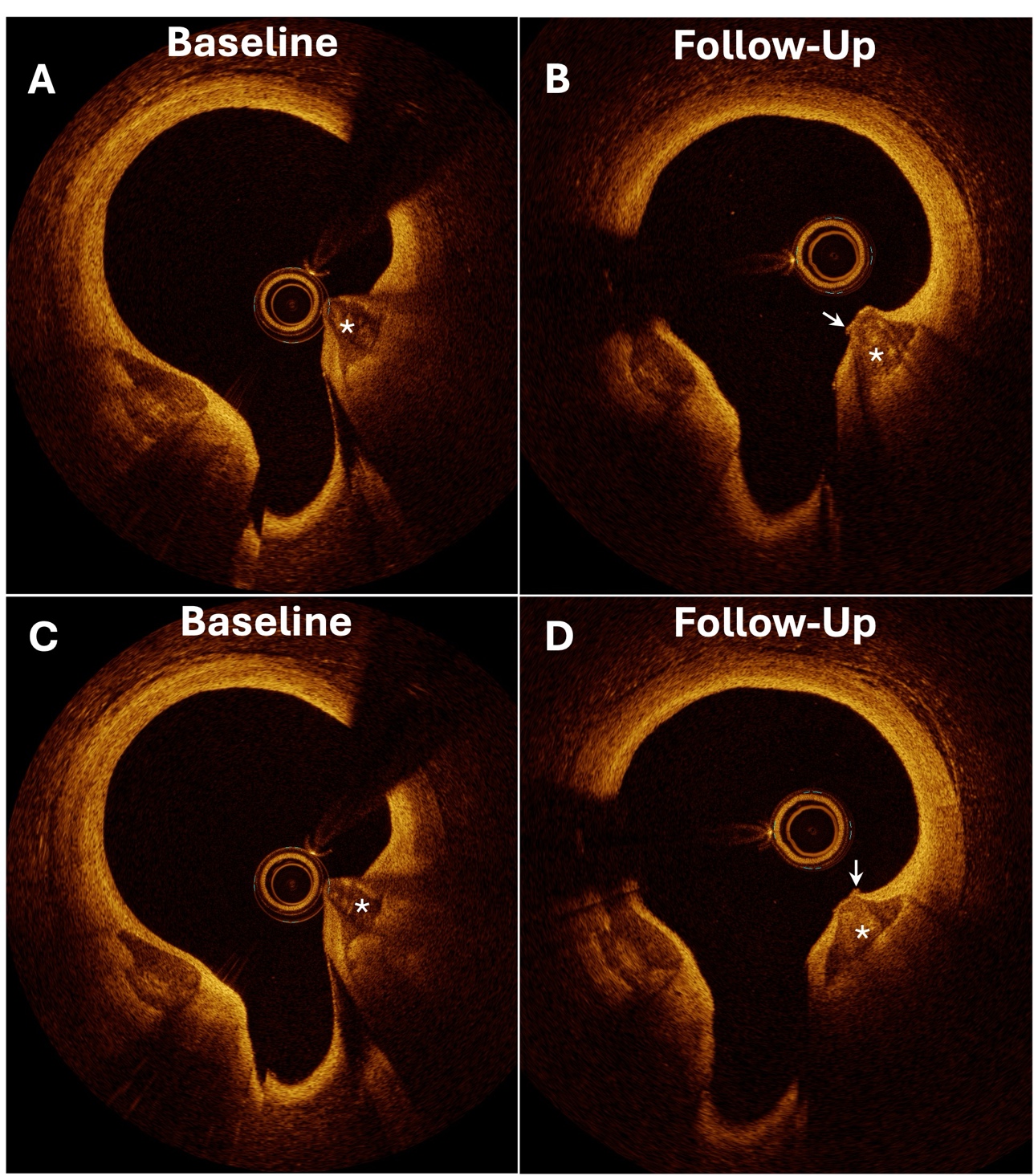


**Supplementary Table 1: Interobserver Reproducibility for Quantitative Measurements**

| **Category** | **Parameter** | **ICC (2,1)** | **95% CI** | **Reliability** |
| --- | --- | --- | --- | --- |
| Calcified Nodule | Minimum FCT | 0.9987 | 0.996-0.999 | Excellent |
| Calcified Nodule | Arc of Calcium | 1 | 1.000-1.000 | Excellent |
| Calcified Nodule | Depth of Calcium | 0.9956 | 0.989-0.998 | Excellent |
| Calcified Nodule | Length of Calcium | 0.9987 | 0.996-0.999 | Excellent |
| Surrounding Calcium | Calcium Arc | 0.9999 | 0.999-1.000 | Excellent |
| Surrounding Calcium | Depth of Calcium | 0.9994 | 0.998-1.000 | Excellent |
| Peak Convexity of CN | Calcium Arc | 0.9975 | 0.994-0.999 | Excellent |
| Peak Convexity of CN | Minimum FCT | 0.998 | 0.995-0.999 | Excellent |
| Peak Convexity of CN | Depth of Calcium | 0.9979 | 0.995-0.999 | Excellent |

CI – Confidence Interval; CN – Calcified Nodule; FCT – Fibrous Cap Thickness; ICC – Intraclass correlation coefficient. Interobserver reproducibility was excellent for all measured parameters, with ICC values ranging from 0.996 to 1.00 (all P<0.001).

**Supplementary Table 2.** Differently expressed genes in patients with CN at baseline.

| Symbol | Expr Log Ratio | p-value | False Discovery Rate (q-value) | Entrez Gene Name |
| --- | --- | --- | --- | --- |
| RNA5SP389 | -29.886 | 1.02E-44 | 3.73E-40 | RNA, 5S Ribosomal Pseudogene 389 |
| MIR5571 | -30 | 4.34E-19 | 7.96E-15 | microRNA 5571 |
| RNA5SP74 | -19.191 | 3.37E-15 | 4.12E-11 | RNA, 5S ribosomal pseudogene 74 |
| **LTF** | 4.647 | 4.37E-11 | 4.00E-07 | lactotransferrin |
| RNA5SP514 | -18.212 | 1.10E-10 | 8.08E-07 | RNA, 5S ribosomal pseudogene 514 |
| RNA5SP134 | -14.289 | 6.41E-09 | 3.92E-05 | RNA, 5S ribosomal pseudogene 134 |
| MAOA | 2.524 | 9.82E-09 | 5.15E-05 | monoamine oxidase A |
| MIR6087 | -5.17 | 4.91E-08 | 2.25E-04 | microRNA 6087 |
| **CD177** | 3.489 | 3.01E-07 | 1.23E-03 | CD177 molecule |
| **CEACAM8** | 3.889 | 6.89E-07 | 2.25E-03 | CEA cell adhesion molecule 8 |
| **DEFA3** | 4.547 | 6.84E-07 | 2.25E-03 | defensin alpha 3 |
| HBBP1 | 16.631 | 7.38E-07 | 2.25E-03 | hemoglobin subunit beta pseudogene 1 |
| **CAMP** | 2.119 | 2.78E-06 | 7.84E-03 | cathelicidin antimicrobial peptide |
| RP11_701P162 | 2.787 | 3.88E-06 | 1.02E-02 | RP11-170N16.2 (Clone-based (Vega) gene) |
| **DEFA4** | 5.018 | 5.34E-06 | 1.31E-02 | defensin alpha 4 |
| RNA5SP481 | -7.582 | 9.94E-06 | 2.28E-02 | RNA, 5S ribosomal pseudogene 481 |
| SNORA49 | -2.682 | 1.22E-05 | 2.64E-02 | small nucleolar RNA, H/ACA box 49 |
| **CEACAM6** | 3.681 | 2.37E-05 | 4.34E-02 | CEA cell adhesion molecule 6 |
| **MMP8** | 3.289 | 2.32E-05 | 4.34E-02 | matrix metallopeptidase 8 |
| RNA5SP225 | -5.378 | 2.36E-05 | 4.34E-02 | RNA, 5S ribosomal pseudogene 225 |
| LINC02289 | 2.783 | 3.72E-05 | 5.94E-02 | long intergenic non-protein coding RNA 2289 |
| RNA5SP161 | -4.839 | 3.45E-05 | 5.94E-02 | RNA, 5S ribosomal pseudogene 161 |
| RPL41P1 | -2.458 | 3.64E-05 | 5.94E-02 | ribosomal protein L41 pseudogene 1 |
| RP11_561P125 | 2.25 | 4.85E-05 | 7.40E-02 | RP11-561P12.5 (Clone-based (Vega) gene) |
| **ARG1** | 2.248 | 7.20E-05 | 9.11E-02 | arginase 1 |
| CNTNAP3 | 2.535 | 6.88E-05 | 9.11E-02 | contactin associated protein family member 3 |
| ITPRIP-AS1 | 1.906 | 7.01E-05 | 9.11E-02 | ITPRIP Antisense RNA 1 |
| **OLFM4** | 3.275 | 6.56E-05 | 9.11E-02 | olfactomedin 4 |
| PLIN4 | 2.249 | 6.27E-05 | 9.11E-02 | perilipin 4 |
| AOC1 | 2.25 | 7.92E-05 | 9.37E-02 | amine oxidase copper containing 1 |
| mir-221 (includes others) | -1.318 | 7.79E-05 | 9.37E-02 | relatives of microRNA 221 |
| AC005050.3 | 1.662 | 8.39E-05 | 9.62E-02 | AC005550.5 (Clone-based (Vega) gene) |
| **LRG1** | 1.622 | 9.21E-05 | 9.78E-02 | leucine rich alpha-2-glycoprotein 1 |
| MAFA | -1.688 | 9.05E-05 | 9.78E-02 | MAF bZIP transcription factor A |
| YPEL3-DT | 1.094 | 9.34E-05 | 9.78E-02 | YPEL3 Divergent Transcript |

**Supplementary Table 3.** Differently expressed genes in PBMC isolated from patients with CN at follow-up

| Symbol | Expr Log Ratio | p-value | False Discovery Rate (q-value) | Entrez Gene Name |
| --- | --- | --- | --- | --- |
| ASZ1 | -30.00 | 5.12E-19 | 3.95E-15 | ankyrin repeat, SAM and basic leucine zipper domain containing 1 |
| **CCL21** | -29.90 | 5.72E-19 | 3.95E-15 | C-C motif chemokine ligand 21 |
| **F9** | 30.00 | 4.93E-19 | 3.95E-15 | coagulation factor IX |
| LOC105374618 | -30.00 | 5.13E-19 | 3.95E-15 | Uncharacterized LOC105374618 affiliated with the ncRNA class |
| RP11-411G2.2 | -30.00 | 5.02E-19 | 3.95E-15 | RP11-560G2.2 (Clone-based (Vega) gene) |
| RP11_343J182 | 30.00 | 4.93E-19 | 3.95E-15 | RP11-343J3.7 (Clone-based (Vega) gene) |
| RP11_687M247 | -29.95 | 5.56E-19 | 3.95E-15 | RP11-187O7.3 (Clone-based (Vega) gene) |
| SNORD114-22 | -29.67 | 1.13E-18 | 6.82E-15 | small nucleolar RNA, C/D box 114-22 |
| RP11_554L122 | -27.73 | 1.79E-16 | 9.61E-13 | RP11-554E23.2 (Clone-based (Vega) gene) |
| LOC124903916 | -27.28 | 5.43E-16 | 2.62E-12 | uncharacterized LOC124903916 |
| DLEU2_3 | -27.12 | 7.85E-16 | 3.44E-12 | Deleted In Lymphocytic Leukemia 2 |
| PTMAP6 | 26.33 | 5.32E-15 | 2.14E-11 | prothymosin alpha pseudogene 6 |
| LOC105378710 | 26.12 | 8.40E-15 | 3.12E-11 | Proline-Rich Extensin-Like Protein EPR1 |
| OR10R1P | 25.34 | 5.20E-14 | 1.79E-10 | olfactory receptor family 10 subfamily R member 1 pseudogene |
| SCARNA20 | 25.10 | 8.98E-14 | 2.89E-10 | small Cajal body-specific RNA 20 |
| RP11_801G162 | 25.01 | 1.09E-13 | 3.28E-10 | RP11-240A16.1 (Clone-based (Vega) gene) |
| AC005692.3 | 24.92 | 1.35E-13 | 3.61E-10 | novel transcrip |
| RP11_734K212 | 24.92 | 1.35E-13 | 3.61E-10 | RP11-734K21.2 (Clone-based (Vega) gene) |
| MTATP6P18 | 23.90 | 1.25E-12 | 3.01E-09 | Mitochondrially Encoded ATP Synthase 6 Pseudogene 18 |
| AC0679601 | 23.38 | 3.71E-12 | 8.51E-09 | novel transcrip |
| AC1047944 | 23.22 | 5.29E-12 | 1.16E-08 | novel transcrip |
| DUXAP6 | -23.10 | 6.69E-12 | 1.40E-08 | double homeobox A pseudogene 6 |
| GOLGA6L6 | -22.14 | 4.50E-11 | 9.04E-08 | golgin A6 family like 6 (gene/pseudogene) |
| RNA5SP152 | -12.07 | 1.68E-09 | 3.23E-06 | RNA, 5S ribosomal pseudogene 152 |
| SLC6A11 | -10.25 | 1.89E-08 | 3.51E-05 | solute carrier family 6 member 11 |
| FYB2 | -18.50 | 3.73E-08 | 6.67E-05 | FYN binding protein 2 |
| RNA5SP202 | -8.12 | 1.08E-07 | 1.86E-04 | RNA, 5S ribosomal pseudogene 202 |
| CEND1P1 | 17.60 | 1.71E-07 | 2.85E-04 | cell cycle exit and neuronal differentiation 1 pseudogene 1 |
| **HBB** | -6.02 | 3.67E-07 | 5.91E-04 | hemoglobin subunit beta |
| RP11_336F141 | 16.77 | 6.33E-07 | 9.86E-04 | RP11-85F14.1 (Clone-based (Vega) gene) |
| CADM2-AS1 | -16.63 | 7.90E-07 | 1.19E-03 | CADM2 antisense RNA 1 |
| RN7SL573P | -3.41 | 9.46E-07 | 1.38E-03 | CADM2 Antisense RNA 1 (Non-Protein Coding) |
| LINC01595 | -15.65 | 3.37E-06 | 4.51E-03 | long intergenic non-protein coding RNA 1595 |
| RNA5SP355 | -3.84 | 3.67E-06 | 4.79E-03 | RNA, 5S ribosomal pseudogene 355 |
| **APPAT** | -1.74 | 7.90E-06 | 1.00E-02 | Atherosclerotic Plaque Pathogenesis Associated Transcript |
| RNU1-24P | -15.02 | 8.20E-06 | 1.01E-02 | RNA, U1 small nuclear 24, pseudogene |
| RNA5SP211 | -6.38 | 8.43E-06 | 1.02E-02 | RNA, 5S ribosomal pseudogene 211 |
| RNA5SP259 | -4.26 | 1.04E-05 | 1.22E-02 | RNA, 5S ribosomal pseudogene 259 |
| LOC124905242 | 14.72 | 1.18E-05 | 1.32E-02 | uncharacterized LOC124905242 |
| RN7SL260P | -3.07 | 1.16E-05 | 1.32E-02 | RNA, 7SL, cytoplasmic 260, pseudogene |
| RNA5SP336 | -12.30 | 1.27E-05 | 1.39E-02 | RNA, 5S ribosomal pseudogene 336 |
| RNA5SP192 | -10.42 | 1.56E-05 | 1.67E-02 | RNA, 5S ribosomal pseudogene 192 |
| CTDNEP1P1 | 1.00 | 1.92E-05 | 2.01E-02 | CTDNEP1 pseudogene 1 |
| CTD_2377D244 | -14.34 | 2.04E-05 | 2.07E-02 | CTD-2377D24.2 (Clone-based (Vega) gene) |
| RNA5SP19 | -5.98 | 2.06E-05 | 2.07E-02 | RNA, 5S ribosomal pseudogene 19 |
| TRPC5OS | -6.08 | 2.28E-05 | 2.25E-02 | TRPC5 opposite strand |
| RNA5SP225 | -4.85 | 2.48E-05 | 2.39E-02 | RNA, 5S ribosomal pseudogene 225 |
| LINC02159 | 14.11 | 2.67E-05 | 2.48E-02 | long intergenic non-protein coding RNA 2159 |
| PPP1R14C | 2.19 | 2.68E-05 | 2.48E-02 | protein phosphatase 1 regulatory inhibitor subunit 14C |
| RPS15AP40 | -2.02 | 2.85E-05 | 2.60E-02 | ribosomal protein S15a pseudogene 40 |
| RASSF6 | -1.41 | 3.50E-05 | 3.08E-02 | Ras association domain family member 6 |
| RNA5SP502 | -12.91 | 3.52E-05 | 3.08E-02 | RNA, 5S ribosomal pseudogene 502 |
| RNA5SP440 | -10.53 | 4.04E-05 | 3.48E-02 | RNA, 5S ribosomal pseudogene 440 |
| C4BPB | -1.42 | 5.66E-05 | 4.79E-02 | complement component 4 binding protein beta |
| RNA5SP286 | -10.78 | 6.94E-05 | 5.77E-02 | RNA, 5S ribosomal pseudogene 286 |
| EPCAM | 2.58 | 8.70E-05 | 7.12E-02 | epithelial cell adhesion molecule |
| RNA5SP338 | -7.33 | 1.02E-04 | 8.20E-02 | RNA, 5S ribosomal pseudogene 338 |
